# Determination of vancomycin and gentamicin clearance in an *in vitro*, closed loop dialysis system

**DOI:** 10.1186/1471-2369-15-204

**Published:** 2014-12-20

**Authors:** Soo Min Jang, Katie E Cardone, Thomas D Nolin, Darius L Mason, Darren W Grabe

**Affiliations:** Department of Pharmacy Practice, Albany College of Pharmacy and Health Sciences, 106 New Scotland Avenue, Albany, 12208 NY USA; Department of Pharmacy Practice, Albany Nephrology Pharmacy Group (ANephRx), Albany College of Pharmacy and Health Sciences, 106 New Scotland Avenue, Albany, 12208 NY USA; Department of Pharmacy and Therapeutics, University of Pittsburgh School of Pharmacy, 3501 Terrace Street, Pittsburgh, 15261 PA USA; Albany College of Pharmacy and Health Sciences, 106 New Scotland Avenue, Albany, 12208 NY USA

**Keywords:** Hemodialysis, *in vitro* system, Vancomycin, Gentamicin, Clearance

## Abstract

**Background:**

The purpose of this study was to evaluate the feasibility of utilizing an *in-vitro*, closed loop hemodialysis system as a method to assess drug clearance. Secondarily, this study tested the influence of variables (blood flow rate, dialysate flow rate, and type of filter) in the hemodialysis procedure on the clearance of vancomycin and gentamicin.

**Methods:**

An *in-vitro*, closed loop hemodialysis system was constructed. The vancomycin (30 mg/L) and gentamicin (25 mg/L) were added to a simulated blood system (SBS). Four conditions (C1-C4) were tested by defining the filter (Polyflux 170H or F180) and the blood and dialysate flow rates (BFR and DFR). All hemodialysis sessions were 3 hours in length and each condition was completed in duplicate. Dialysate effluent was collected in a 50 gallon polyethylene drum. Samples were collected (in duplicate) from the SBS and the dialysate effluent at baseline and at the end of the hemodialysis session. Samples were analyzed for vancomycin and gentamicin with an ultrahigh performance liquid chromatography/tandem mass spectrometry method.

**Results:**

A total of eight 3-hour hemodialysis sessions were conducted. For all tested conditions (C1-C4), vancomycin was undetectable in the SBS at the end of dialysis. However, total vancomycin recovery in the dialysis effluent was 85±18%, suggesting that up to 15% may have adsorbed to the dialysis filter or tubing. Gentamicin clearance from SBS was >98% in all tested conditions. Average gentamicin recovery in the dialysate effluent was 99±15%.

**Conclusion:**

Both vancomycin and gentamicin were readily removed by high-flux hemodialysis under all conditions studied. No significant differences in drug clearance were observed between conditions used in this *in vitro* study. The clinical implications of changing these hemodialysis parameters are unknown.

## Background

Pharmacokinetics are significantly altered during hemodialysis and require directed studies in this setting in order to guide drug dosing [1, 2]. These clinical studies provide valuable information but are limited since dialysis variables (flow rates, filter selection, time on dialysis) may or may not be standardized [3–11]. Studies can be designed using one regimen with standardized blood flow rates (BFR) and dialysate flow rates (DFR), similar hemodialysis filters and similar convective (ultrafiltration) flow rates (UFR). While this type of design reduces variability and is desirable in a small sample size, it may confine application of the results to that stated hemodialysis prescription and thus the determined pharmacokinetic parameters. Studies that do not standardize the dialysis prescription lack clarity on the potential influence of those variables on drug clearance. A more comprehensive study to evaluate the impact of a change in one or more of these parameters is desirable but challenging to conduct in an *in vivo* design. However, these studies may be feasible in a simulated model of hemodialysis. While *in vitro* models of dialysis have been used to predict drug clearance, most have investigated the influence of changing just one variable such as the dialyzer [12, 13].

This study was aimed at characterizing the clearance of vancomycin and gentamicin and the influence of changes in 3 variables (BFR, DFR and hemodialysis filters) on clearance in a closed loop *in vitro* dialysis system. It is hypothesized that the clearance of vancomycin and gentamicin will increase with increasing flow rates and more efficient hemodialysis filters.

## Methods

### Study design

This was an *in vitro* pharmacokinetic study of two antibiotics in a model of hemodialysis.

### Description of model

A closed-loop fixed volume reservoir of a 5-L normal saline solution (4750 mL reservoir +250 mL tubing and dialysis membrane volume) was prepared for a simulated blood system (SBS). The normal saline had a pH of 5.0 and an osmolarity of 308 mOsmol/L. Polyvinyl chloride tubing was used to create the SBS. The supply lines were connected from the dialysate supply [Acid (NaturaLyte Liquid Acid Concentrate 2 mEq/L potassium, 2.5 mEq/L calcium) and bicarbonate powder (NaturaLyte; Fresenius)] to the filter and from the filter into a 50 gallon plastic drum for collection of the dialysate. The system was primed with 250 mL of normal saline prior to initiation of the dialysis procedure and maintained at 37°C.

Four different conditions were studied (Table 1): two sets of BFR (400 mL/min and 500 mL/min) and DFR (600 mL/min and 800 mL/min) were tested. Table 1 also outlines the characteristics of the two hemodialysis membranes that were used in the study [Polyflux 170H (Gambro) and the Optiflux F180 (Fresenius)]. The UFR was maintained at 300 mL/hr to approximate the net fluid removal of one liter, to simulate a typical hemodialysis prescription, and to minimize convective solute clearance. A volumetric HD machine (model 2008H, Fresenius USA, Walnut Creek, CA) was used to control the BFR, DFR and UFR. Each hemodialysis session was conducted for 3 hours with no interruption. The dialysate solution was flushed counter-current to SBS within the dialysis membrane at the end of the session to rinse out tubing. Each HD procedure was conducted in duplicate.

**Table 1:** **Dialysis conditions tested**

Conditions	Blood flow rate (mL/min)	Dialysate flow rate (mL/min)	Filter	Filter characteristics
C1	400	600	Polyflux (Gambro)	Surface area 1.7 m^2^; Wall thickness 50 microns; Inner diameter 215 microns; Ultrafiltration coefficient 70^*^
C2	500	800	Polyflux (Gambro)	
C3	400	600	Optiflux F180 (Fresenius)	Surface area 1.8 m^2^; Wall thickness 40 microns; Inner diameter 200 microns; Ultrafiltration coefficient 60^+^
C4	500	800	Optiflux F180 (Fresenius)	

*Measured with bovine blood, Hematocrit = 32%, Protein 60 g/L, at 37°C.

+Measured with *in vitro* bovine, Hematocrit = 32%.

### Sample preparation and collection

Vancomycin 150 mg and gentamicin 125 mg were injected into the SBS to achieve final concentrations of 30 mg/L and 25 mg/L, respectively. Samples (5 mL) were collected from SBS and dialysate at 0 and 180 min (end of session). All spent dialysate during the simulated HD session was collected in a 50 gallon drum and mixed prior to sampling (5 mL aliquot) for vancomycin and gentamicin. Collected samples were labeled and stored at -70°C until analysis. Collected samples were stored in Nalgene freezer vials and shipped in dry ice for analysis.

### Sample analysis

Vancomycin and gentamicin concentrations in study samples were simultaneously determined using an ultra-high performance liquid chromatography/tandem mass spectrometry (UHPLC-MS/MS) method with modifications of Li *et al*. [14] Briefly, 25 μL of sample was diluted with 20 mM pentafluoropropionic acid (PFPA, 250 μL), then injected (10 μL) directly onto the LC-MS/MS. Separation was achieved using a Thermo Accela UHPLC system with a Thermo Hypersil GOLD C18 (50 X 2.1 mm, 1.9 μm) column, and a 5 mM PFPA in water to 5 mM PFPA in acetonitrile gradient. Detection and quantification of analytes was achieved with a Thermo TSQ Quantum Ultra triple quadrupole mass spectrometer via heated electrospray ionization in positive ionization mode. Selective reaction monitoring was utilized to follow precursor to product ion *m/z* transitions of 724➜144 for vancomycin, 450➜322 for gentamicin C1a, 464➜322 for gentamicin C2 + C2a, 478➜322 for gentamicin C1, 468➜163 for tobramycin (internal standard for gentamicin), and 646➜290 for cefoperazone (internal standard for vancomycin). The calibration curves for the analysis ranged from 0.5 μg/mL (the lower limit of quantification) to 35.0 μg/mL for both analytes. The intra- and inter-day precision and accuracy were ≤12%.

### Data analysis

The mean ± standard deviation (SD) vancomycin and gentamicin concentrations were calculated for each sample. The percent change in vancomycin and gentamicin concentration from beginning to end of HD session and absolute amount of vancomycin and gentamicin eliminated were calculated. A mean loss of greater than 15% from the SBS of the initial concentration was considered as a clinically important loss of either vancomycin or gentamicin. Drug recovery was estimated using the following mass balance equation:


DFR: Dialysis flow rate (mL/min).

t: Hemodialysis duration (minutes).

C_D_: The drug concentration in dialysate (μg/mL).

To calculate hemodialysis clearance (CL_D_) [15]:


C_D_: The drug concentration in dialysate (μg/mL).

Vol _D_: The total volume of dialysate collected during the dialysis time (mL).

A: Average concentration of drug in plasma entering the dialyzer (μg/mL).

t: Hemodialysis duration (minutes).

Dialysis sessions were conducted in the ANephRx Core laboratory at the Albany College of Pharmacy and Health Sciences. Vancomycin and gentamicin concentrations were analyzed at the University of Pittsburgh.

### Statistical analysis

Data are reported as mean ± standard deviation. Comparisons of filters intradialytic clearances were made using the Student’s t-test except for the C3 condition (Polyflux BFR 400 mL/min; DFR 600 mL/min) where UFR was different (UFR = 250 mL/hr) from other conditions (UFR = 300 mL/hr). All statistical tests were performed as two-sided, and a P < 0.05 was considered significant.

## Results

A total of eight 3-hour hemodialysis sessions were conducted. Instillation of both vancomycin and gentamicin into the SBS system resulted in a clear solution. The dialysate compartment also consisted of a clear solution. There was no visual evidence of color change in the SBS or dialysate compartments during the study period.

Table 2 outlines the drug concentrations at different time intervals (pre- and post-dialysis session) in four experimental conditions. Table 3 summarizes the quantity of vancomycin and gentamicin recovered in dialysate after a 3-hour dialysis session using the mass balance equation and the two drugs’ hemodialytic clearances. The mean vancomycin hemodialysis clearances (mL/min) were 82.1, 53.3, 64.1 and 47.7 respectively for conditions through C1 to C4. The mean quantity recovered in dialysate was 142.6 ± 9.6 mg and was undetectable in the SBS at the end of dialysis for all tested conditions (C1-C4), indicating complete removal from the SBS compartment. The mean gentamicin hemodialysis clearances (mL/min) were 58.2, 43.5, 56.7 and 40.6 respectively for C1 to C4. The mean quantity recovered in dialysate was 135.7 ± 8.9 mg. Drug recovery in the effluent was 85±18% for vancomycin and 99±15% for gentamicin. Vancomycin and gentamicin clearances in C2 and C4 (BFR of 500 mL/min and DFR of 800 mL/min) were similar. Specifically, vancomycin clearances with Polyflux and Optiflux filters were 53.3 and 47.7 mL/min, respectively (p = 0.3), and gentamicin clearances were 43.5 and 40.6 mL/min, respectively (p = 0.6). Vancomycin and gentamicin clearances were also similar in C1 (BFR 400 mL/min and DFR 600 mL/min) and C2 (BFR 500 and DFR 800 ml/min) in Optiflux filter. Vancomycin clearances were 82.1 and 53.3 ml/min, respectively (p = 0.4). Gentamicin clearances in C1 and C2 were 58.2 and 43.5 ml/min, respectively (p = 0.1).

**Table 2:** **The concentration of vancomycin and gentamicin at different time interval in different conditions**

Condition	Filter	Sol	Time (min)	BFR (mL/min)	DFR (mL/min)	Vanco (ug/mL)	TG (ug/mL)
C1	Poly	SBS	0	400	600	20.04 ± 0.86	26.65 ± 1.69
	Poly	SBS	180	400	600	BLQ	0.51 ± 0
	Poly	Dial	0	400	600	BLQ	BLQ
	Poly	Dial	180	400	600	1.35 ± 0.41	1.31 ± 0.18
C2	Poly	SBS	0	500	800	31.91 ± 2.5	35.93 ± 0.13
	Poly	SBS	180	500	800	BLQ	0.53 ± 0.02
	Poly	Dial	0	500	800	BLQ	BLQ
	Poly	Dial	180	500	800	1.06 ± 0.05	0.99 ± 0
C3	Opt^*^	SBS	0	400^*^	600^*^	23.74 ± 5.27	25.41 ± 3.47
	Opt	SBS	180	400	600	BLQ	0.52 ± 0.01
	Opt	Dial	0	400	600	BLQ	BLQ
	Opt	Dial	180	400	600	1.20 ± 1	1.14 ± 0.93
C4	Opt	SBS	0	500	800	30.75 ± 0.43	36.97 ± 0.18
	Opt	SBS	180	500	800	BLQ	0.51 ± 0
	Opt	Dial	0	500	800	BLQ	BLQ
	Opt	Dial	180	500	800	0.91 ± 0.08	0.94 ± 0.15

*UFR is constant at rate of 300 ml/hr except for the one condition.* ^*^UFR was 250 mL/hr. Sol: Solution; Poly: Polyflux filter; Opt: Optiflux filter; Dial: Dialysate; Vanco: Mean vancomycin concentration; TG: Mean total gentamicin concentration; BLQ: Below the lower limit of quantification.

**Table 3:** **Summary of the quantity of vancomycin and gentamicin using the mass balance equation and dialytic clearance**

Condition	Filter	Sol	BFR (mL/min)	DFR (mL/min)	Vanco (mg)	Gent (mg)	Cl _Vanc_(mL/min)	Cl _Gent_(mL/min)
C1	Poly	Dial	400	600	147.0 ± 44.7	142.7 ± 20	82.1	58.2
C2	Poly	Dial	500	800	152.9 ± 7.2	142.7 ± 1.02	53.3	43.5
C3	Opt^*^	Dial	400^*^	600^*^	131.0 ± 108.4^*^	124.0 ± 101.5^*^	65.3^*^	56.6^*^
C4	Opt	Dial	500	800	131.9 ± 12.3	137.0 ± 21.5	47.7	40.6

*UFR is constant at rate of 300 ml/hr except for the one condition.* ^*^UFR was 250 ml/hr. Sol: Solution; Poly: Polyflux filter; Opt: Optiflux filter; Dial: Dialysate; Vanco: Vancomycin concentration; Gent: Total Gentamicin concentration; BLQ: Below the lower limit of quantification; Cl_Vanc_: Mean Vancomycin Clearance; Cl_Gent_: Mean Gentamicin clearance.

## Discussion

To our knowledge, this is the first study to investigate the influence of changes in BFR, DFR, and two different filters (Polyflux and Optiflux) on vancomycin and gentamicin clearance using an *in-vitro* closed loop dialysis system. Interestingly, regardless of types of filter, the rate of BFR and DFR, vancomycin was undetectable in the SBS at the end of dialysis for all tested conditions (C1-C4), suggesting the drug is readily cleared by contemporary hemodialysis. Gentamicin also exhibited nearly complete removal. However, gentamicin was consistently measured in the post-dialysis SBS with the mean value of 0.52 ± 0.01ug/mL. This was unexpected since the molecular weight of vancomycin (1485.74 g/mol [16]) is larger than the molecular weight of gentamicin C1 (477.6 g/mol [17]).

Eighty five percent (±18%) of vancomycin was recovered in the dialysis effluent compared to 99±15% with gentamicin. Drug characteristics such as molecular size, steric hindrance, can affect the clearance, and the 15% difference suggests that some vancomycin may have adsorbed to the dialysis filter or tubing [18]. The contribution of ultrafiltration to the overall clearance of vancomycin and gentamicin was unable to be ascertained since the ultrafiltration rate of 300 mL/hr was held constant. The UFR may affect hemofiltration (drug elimination) [19] yet, it did not in our study. A limitation of this study was the use of normal saline instead of blood in the SBS and a one compartment model. Additionally, filters were not evaluated to verify and quantify vancomycin filter binding. The study results cannot be used to predict drug pharmacokinetics and pharmacodynamics but rather the potential for drug clearance as it pertains to changes in a dialysis prescription. This knowledge could be helpful if used in concert with available clinical data. If drug clearance can be demonstrated in such a model, clinical trials may not be necessary (presuming no active metabolites). In one study, the reliability of an *in vitro* model of vancomycin clearance to predict drug clearance was demonstrated [20]. There have been few *in-vitro* studies with other medications such as intravenous iron sucrose and dextran [21, 22]. Pinner et al. determined that the *in vitro* method overestimated *in vivo* dialysis clearance of vancomycin and gentamicin, 27% and 17%, respectively [13]. However, only one set of dialysis parameters was studied, which is an important distinction of that study compared to the present investigation [13].

## Conclusion

This study aimed to characterize the clearance of vancomycin and gentamicin and the influence of changes in 3 variables (BFR, DFR and hemodialysis filters) on clearance in a closed loop *in vitro* dialysis system. Our study did not detect a difference in the clearance of vancomycin and gentamicin with increasing flow rates and more efficient hemodialysis filters. The concentrations of vancomycin and gentamicin in the SBS were undetectable at the conclusion of most sessions. This reflects nearly 100% clearance of both molecules in all sessions. The observed differences in the clearance of vancomycin and gentamicin between clinical studies and this study were likely due to the absence of protein binding and other volume of distribution changes not reflected in the *in vitro* system. Adaptation of the model to reflect *in vivo* characteristics may improve the performance of the system to detect differences in solute clearance. In addition, this underscores the importance of clinical pharmacokinetic and pharmacodynamic studies to validate data obtained from *in vitro* studies.

## Abbreviations

SBS: Simulated blood system
C1: Condition 1
C2: Condition 2
C3: Condition 3
C4: Condition 4
BFR: Blood flow rate
DFR: Dialysate flow rate
UFR: Ultrafiltration flow rate
HD: Hemodialysis
UHPLC: Ultra-high performance liquid chromatography
MS: Mass spectrometry
PFPA: Pentafluoropropionic acid
SD: Standard deviation.
